# Galectin-3 and Its Genetic Variation rs4644 Modulate Enterovirus 71 Infection

**DOI:** 10.1371/journal.pone.0168627

**Published:** 2016-12-21

**Authors:** Wen-Chan Huang, Hung-Lin Chen, Huan-Yuan Chen, Kuan-Po Peng, Yungling Lee, Li-Min Huang, Luan-Yin Chang, Fu-Tong Liu

**Affiliations:** 1 Institute of Biomedical Sciences, Academia Sinica, Taipei, Taiwan; 2 Department of Pediatrics, National Taiwan University Hospital and College of Medicine, National Taiwan University, Taipei, Taiwan; 3 Ph.D. Program in Translational Medicine, National Taiwan University and Academia Sinica, Taipei, Taiwan; 4 School of Medicine, National Yang-Ming University, Taipei, Taiwan; 5 Institute of Epidemiology and Preventive Medicine, National Taiwan University, Taipei, Taiwan; 6 Department of Dermatology, University of California Davis, Sacramento, California, United States of America; University of Hong Kong, HONG KONG

## Abstract

Galectin-3, a chimeric type β-galactoside-binding protein, is known to modulate viral infection; however, its role in enterovirus 71 (EV71) infection has not been investigated. We generated galectin-3 null rhabdomyosarcoma (RD) cells and evaluated whether EV71 infection would be affected. In galectin-3 null cells, the released and intracellular EV71 viral loads were suppressed after 24 h of infection, and cell death rates were significantly lower, while cell proliferation remained unaltered. In addition, RD cells expressing a nonsynonymous genetic variant of galectin-3, rs4644 (*LGALS3* +191C/A, P64H), produced lower virus titers than those with wild-type galectin-3 (C allele). To clarify whether the *in vitro* viral load reduction correlates with clinical severity, we enrolled children with laboratory-confirmed EV71 infection. Since hyperglycemia is an indicator of severe EV71 infection in children, 152 of 401 enrolled children had glucose examinations at admission, and 59 subjects had serum glucose levels ≥ 150 mg/dL. In comparison to the rs4644 AA genotype (2.2 ± 0.06 log_10_ mg/dL), serum glucose levels during EV71 infection were higher in patients with CC (2.4 ± 0.17 log_10_ mg/dL, *p* = 0.03) and CA (2.4 ± 0.15 log_10_ mg/dL, *p* = 0.02) genotypes, respectively. These findings suggest that the rs4644 AA genotype of galectin-3 might exert a protective effect. In summary, galectin-3 affects EV71 replication in our cellular model and its variant, rs4644, is associated with hyperglycemia in the clinical setting. The underlying mechanism and its potential therapeutic application warrant further investigation.

## Introduction

Encephalitis and cardiopulmonary failure are the most critical complications occurring in young children or in immunocompromised hosts with EV71 infections [1,2]. In tertiary center studies, approximately 10% of all EV71 patients presented with these severe complications, with a mortality rate of up to 40% in children with cardiopulmonary failure [3,4]. In children, hyperglycemia is one predictor for severe EV71 infection, particularly in those with cardiopulmonary failure, and associated with worse clinical outcomes [1,5]. The pathogenesis of hyperglycemia is still debated; excessive sympathetic hyperactivities induced by severe EV71 infection is considered a possible mechanism of impaired glucose regulation [6].

EV71 infection and its complications are associated with certain haplotypes of human leukocyte antigens and cytokine pertinent genetic polymorphisms [7–10]. However, intracellular proteins were also known to regulate host responses to viral infection. For example, galectin-3, a β-galactoside-binding lectin, is present in various cell types and tissues, and modulates immune reaction against pathogen invasion and regulates cellular homeostasis, e.g., cell growth, apoptosis, and glucose metabolism [11–14]. Increased expression of galectin-3 has been reported in tissues collected from patients with hepatitis B virus and hepatitis C virus infections [15,16]. Galectin-3 also promotes virus budding of human immunodeficiency virus [17]. Among the genetic variants of galectin-3, rs4644 is one of the two major single nucleotide polymorphisms (SNPs) with minor allele frequency of > 5%. This genetic variant is a nonsynonymous SNP (c.191C>A) with a missense mutation (p.P64H). The data from the HapMap project indicated that the minor allele (A allele) frequency of rs4644 in Chinese ethnicity was 22%, corresponding to genotype frequencies of CC (58%), CA (40%), and AA (2%), respectively [18]. Rs4644 possibly modulates certain cancer susceptibility: AA genotype were more frequent in breast cancer patients in both Western and Asian populations [19]. Furthermore, an European study revealed that the elderly carriers of rs4644 AA genotype had higher levels of C-reactive protein (CRP) [20]. These results suggest that this genetic variant of galectin-3 may be associated with inflammatory status in human diseases. In this study, we intended to evaluate whether galectin-3 and its variant, rs4644, affect EV71 infection in both laboratory and clinical settings.

## Materials and Methods

### In vitro exploration

#### Cell culture with viral infection

Human rhabdomyosarcoma (RD) cells were grown in Dulbecco’s modified Eagle medium (DMEM) with 10% fetal bovine serum (FBS) and 1% penicillin-streptomycin (Gibco, Gaithersburg, MD, USA) at 37°C. EV71 (TW/4643/98) virus infection was performed in serum-free DMEM for 1 h at 37°C. The virus-infected cells were washed twice in phosphate-buffered saline (PBS) and then cultured in DMEM containing 2% FBS at 37°C. The medium was collected 72 h post infection and the cells were lysed by three freeze-thaw cycles. The supernatant containing EV71 virus was collected by centrifugation at 3,000 *g* for 30 min at 4°C to discard the cell debris. The virus was concentrated with 30% sucrose cushion by ultracentrifugation using Beckman SW28 rotors (Beckman Coulter, Brea, CA, USA) at 121,896 *g* (26,000 rpm) for 4h at 4°C. To determine viral titers in each sample or virus stock, RD cells were seeded in 6-well plates for plaque assays. These cells were infected with 10-fold serial dilutions of virus samples in serum-free DMEM. After incubation for 1 h, the medium was replaced with 2 ml DMEM containing 2% FBS and 0.3% agarose. Three days after incubation at 37°C under 5% CO_2_, cells were fixed using 3.7% formalin (Sigma-Aldrich, St. Louis, MO, USA) and stained with 0.05% crystal violet. Pan-caspase inhibitor, z-vad-fmk (Selleckchem, Houston, TX, USA), was added to the medium during the entire course of EV71 infection in experiments requiring apoptosis inhibition.

#### Generation of galectin-3 knock-out (G3KO) RD cells

**Fig 1:**
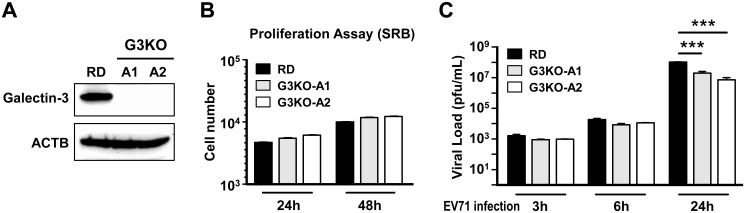
Galectin-3 promotes EV71 infection. (A) Using TALEN, ablation of the *galectin-3* gene was performed in RD cells. Two clones of galectin-3 knock-out (G3KO) RD cells were selected. Protein expression of galectin-3 was examined in cell lysates from RD cells and two G3KO clones (G3KO-A1 and G3KO-A2) by immunoblotting. (B) RD and G3KO cells were seeded in 96-well plates. Cell proliferation was evaluated at 24 and 48 h by SRB assays. (C) RD and G3KO cells were seeded in 6-well plates and were infected with EV71 (MOI 0.1). After 3, 6 and 24 h, virus titers in culture supernatants and infected cells were examined by plaque assays. Assays were carried out in triplicates per cell type and normalized to the numbers of live cells. ACTB, beta-actin. ***P < 0.001.

G3KO RD cells were generated using transcription activator-like effector nucleases (TALEN) shown in Fig 1A. TALEN constructs of galectin-3 were designed by TAL Effector Nucleotide Targeter 2.0 (Cornell University, USA) [21]. Golden Gate TALEN and TAL Effector Kit (Addgene, Cambridge, MA, USA) were used to assemble the TALEN plasmids according to the manufacturer’s protocol. TALEN vectors were transfected into RD cells using Effectene Transfection Reagent (Qiagen, Hilden, Germany). Twenty-four hours after transfection, single colonies were selected by flow cytometry and seeded in 96-well plates. Cells from single colony selection were screened using a T7 endonuclease assay. G3KO RD cells were verified by DNA sequencing and immunoblotting, and wild-type RD cells were used as controls.

#### Cell proliferation assay

Cell proliferation was determined by sulforhodamine B (SRB) assays. Serial dilutions of cells were seeded in 100 μL complete medium in 96-well microtiter plates. After 24 and 48 h of incubation, the medium was removed and 50 μL 10% trichloroacetic acid (4°C) was added in each well for 1 h. The microtiter plates were then rinsed with water 5 times. After removing the wash residue in the plates, 100 μL SRB solution (0.4% w/v SRB dissolved in 1% acetic acid) was added to each well and incubated for 30 min. SRB solution was then removed from the plates by washing with 1% acetic acid 5 times. The culture plates were air-dried until no moisture was visible. To solubilize the dye, 100 μL 10 mM Tris base was added to each well, and absorption at 564 nm was measured.

#### Cell viability assay

Cell viability was determined using MTT (3-(4,5-dimethylthiazol-2-yl)-2,5-diphenyltetrazolium bromide) assays. Cells (4,000/well) were seeded in 96-well plates and incubated overnight. The cells were then incubated in FBS-free DMEM culture medium with or without EV71 (MOI 0.1). After 1 h of incubation, the medium was replaced with DMEM containing 2% FBS. MTT was added after 3, 6, 18, or 24 h post infection, and incubated for 30 min. The medium was then removed and DMSO was added to each well. Absorption at 570 nm was determined. Values for EV71-induced events were subtracted from values for the control, and the results were normalized to control levels of MTT reduction. Thus, the scale of MTT reduction represented the cell death caused by EV71 infection (8 replicates per cell type).

#### Overexpression of wild-type galectin-3 and rs4644 AA genotype in G3KO RD cells

Detailed methods for acquiring full-length galectin-3 cDNA were described in previous studies [17,22]. Two types of galectin-3 cDNA were subcloned into pEGFP-N1 vector (Clontech, Mountain View, CA, USA): wild-type galectin-3 (G3-WT) and rs4644 A allele (G3-64A), to mimic wild-type galectin-3 and rs4644 AA genotype respectively. In 6-well plates, G3KO RD cells were transfected with the two types of galectin-3 or vector control using Effectene transfection reagent (Qiagen, Hilden, Germany).

#### Immunoblotting

Cells were lysed using a solution containing 100 mM NaCl, 300 mM Tris (pH 7.4), 1% NP-40, 1% sodium deoxycholate, 1% SDS, 0.5 mM EDTA, and proteinase inhibitor cocktail (Merck Millipore, Billerica, MA, USA). Lysates were sonicated and incubated on ice for 10 min before centrifugation at 16,000 *g* for 10 min. Proteins in the lysates were separated by 12% SDS-PAGE and transferred to PVDF membranes. The membranes were probed with anti-EV71 (#Mab979, Millipore, Billerica, MA, USA), anti-galectin-3 (produced in-house, and purified by GeneTex, Irvine, CA, USA), PARP (#9542, Cell Signaling, Danvers, MA, USA), caspase-3 (#9665, Cell Signaling, Danvers, MA, USA), and anti-beta-actin (#A5441, Proteintech, Manchester, UK).

### In vivo exploration

#### Patients and disease definition

Between May 1999 and November 2012, children under 4 years of age and presenting symptoms of enterovirus infection, e.g. herpangina or hand-foot-and-mouth disease, were enrolled in the present study at six hospitals in Taiwan, including National Taiwan University Hospital, Taipei; Chang Gung Memorial Hospital, Taoyuan; Children’s Hospital, China Medical University and Hospitals, Taichung; Changhua Christian Hospital, Changhua; National Taiwan University Hospital, Yun-Lin Branch, Yunlin; Kaohsiung Chang Gung Memorial Hospital, Kaohsiung. This study was approved by the institutional review board in each hospital. Written informed consents were duly obtained from parents or guardians of the patients, by following the regulations of the institutional review board in each hospital. All children enrolled had laboratory-confirmed EV71 infections. The severity of EV71 infection was classified as follows: stage 1, hand-foot-and-mouth disease or herpangina; stage 2, meningitis or myoclonic jerk; stage 3A, encephalitis with autonomic dysfunction; stage 3B, encephalitis with cardiopulmonary failure.

#### Identification of clinical isolates

Two throat swabs were collected from each patient for virus isolation and molecular examination by real-time reverse transcription polymerase chain reaction (RT-PCR) followed by VP1 gene, which encodes the major structural protein of EV71. If the symptoms persisted for more than 4 days before enrollment, two rectal swabs were collected for viral examination. Detailed methods and materials were described in our previous studies [23,24]. Confirmation of EV71 infection was based on positive results from virus isolation and/or positive EV71 real-time RT-PCR with VP1 sequencing.

#### Allelic discrimination assay

Genomic DNA was isolated from the patients’ peripheral blood mononuclear cells by standard procedures. Taqman^®^ SNP genotype assay (Applied Biosystems, Foster City, CA, USA) was used to identify the genotype of *LGALS3* +191. Detailed methods are described in previous reviews [25,26].

#### Statistical analysis

Clinical results were analyzed using SPSS version 20 (IBM, New York, NY, USA). Quantitative results obtained from functional assays were analyzed using Prism 6 software (GraphPad Software, La Jolla, CA, USA). A one-way analysis of variance (ANOVA) with Tukey’s post-hoc test was used to analyze differences among groups. Differences were considered statistically significant at *p* values < 0.05.

## Results

### Galectin-3 modulates EV71 replication in RD cells

Cell proliferation rates were similar between RD cells and G3KO clones, when they were cultured for 24 and 48 h (Fig 1B and S1A Fig). We then infected RD and G3KO cells with EV71 and checked viral loads at 3, 6 and 24 h after infection. No significant changes between groups were observed at 3 and 6 h post infection [p.i.], as determined by viral titers measured in the culture supernatant and cell lysates. However, wild-type RD cells produced much higher viral loads at 24 h p.i., compared with G3KO clones (Fig 1C). We further analyzed the viral titers in the supernatant and cell lysates respectively. Between 3 and 18 h of EV71 infection, the intracellular viral loads did not significantly differ between RD and G3KO clones (S1C Fig). Yet, at 24 h p.i., both the released and intracellular viral loads were higher in RD cells than in G3KO cells (S1B and S2C Figs).

### Galectin-3 expression adversely affects cell viability during EV71 infection

**Fig 2:**
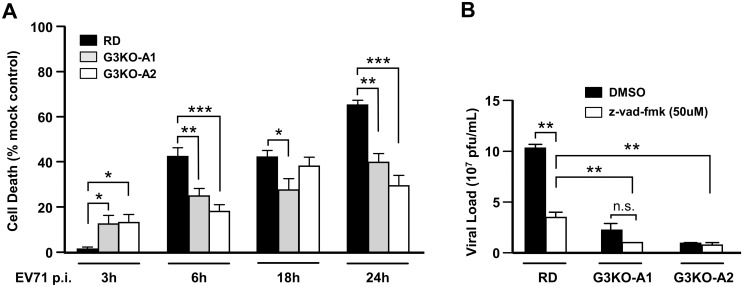
Galectin-3 is involved in cell viability in EV71 infection, which is partly-independent of caspase-induced apoptosis. (A) RD and G3KO cells (A1 and A2 clones) were seeded in 96-well microtiter plates. Using MTT assay, cell viability was examined 3, 6, 18, and 24 h post EV71 infection. Values for EV71-induced events were subtracted from values of control, and results were scaled to control levels of MTT reduction. (B) RD and G3KO cells were infected with EV71 (MOI 0.1) for 24 h. The medium contained a pan-caspase inhibitor (z-vad-fmk, 50 μM) or control (DMSO) during the 24 h period. Culture supernatants and cell lysates were used to carry out plaque assays to determine viral titers (triplicates per cell type). *P < 0.05, **P < 0.01, ***P < 0.001, n.s., not significant.

To understand whether galectin-3 influences cell survival in EV71 infection, we first observed the kinetics of cell viability during EV71 infection by MTT assay (Fig 2A). At the initial phase of infection (3 h p.i.), G3KO cells exhibited a higher death rate than wild-type cells. However, at 6, 18, and 24 h p.i., cell death rates in G3KO cells were either lower than or similar to that in wild-type RD cells. The results indicate that galectin-3 affects cell survival differently at early and late phases of EV71 infection. To further evaluate the effect of galectin-3 on EV71-induced apoptosis, we examined the expressions of apoptosis-related proteins with immunoblots and ELISA at 24 h p.i. Both the activities of PARP and caspase-3 were higher in RD cells after EV71 infection, compared with G3KO cells (S2A and S2B Fig). It indicates that galectin-3 ablation suppresses EV71-induced apoptosis.

In order to examine whether galectin-3 modulates EV71 release through caspase-dependent apoptosis, we measured the total viral titers and intracellular viral loads, with or without a pan-caspase inhibitor. In RD cells, the total viral titers were decreased after caspases were inhibited at 24 h p.i. (Fig 2B), but the intracellular viral loads were unaffected (S2C Fig). In G3KO cells, the total and the intracellular viral productions were not changed when apoptosis was inhibited. The results suggest that galectin-3 modulates EV71 release through apoptosis. Moreover, under the suppression of apoptosis, we observed the total (Fig 2B) and the intracellular viral loads (S2C Fig) were still higher in RD cells than in G3KO cells at 24 h p.i. Thus, galectin-3 modulates EV71 infection partly through caspase-independent apoptosis.

### Rs4644 A allele is associated with lower EV71 viral titers

**Fig 3:**
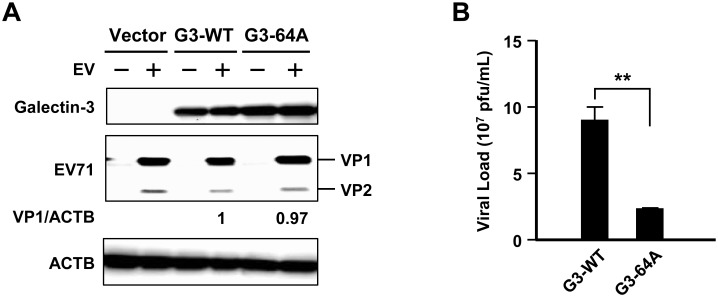
Cells expressing the rs4644 A allele had lower EV71 viral loads. G3KO RD cells were transfected with plasmids encoding EGFP (vector control), wild-type galectin-3 (G3-WT), and rs4644 A allele (G3-64A). The cells were infected with EV71 for 24 h. Expression of galectin-3 and viral proteins were assessed using immunoblotting (A). EV71 viral titers in cell lysates and culture supernatant were determined by plaque assays (B). Plaque assays were carried out in triplicates per cell type.

In addition to G3KO cells, we examined the effects of galectin-3 SNP rs4644 on EV71 replication in RD cells. Plasmids encoding the two types of galectin-3, wild-type C allele galectin-3 (G3-WT) or rs4644 A allele (G3-64A) were transfected into G3KO cells, and the following day, the cells underwent EV71 infection for 24 h. EV71 proteins and ectopic expression of galectin-3 are illustrated in Fig 3A. Cells with G3-WT had significantly higher viral loads than those with G3-64A (Fig 3B).

### Clinical associations between galectin-3 SNP rs4644 and disease severity in children with EV71 infection

To evaluate the clinical associations between rs4644 and disease severity in EV71 infection, 401 children under 4 years of age were enrolled in the present study. Table 1 shows the demographic data of the individuals enrolled. The male to female ratio was 1.6, and the mean age was 1.9 ± 1.0 years. Patients were classified into four groups according to the clinical severity of infection: stage 1, 108 (27%); stage 2, 207 (52%); stage 3A, 45 (11%); and stage 3B, 41 (10%). All stage 1 and 2 patients completely recovered from their illnesses. Only patients with encephalitis (stages 3A and 3B) had neurological sequelae (41/401, 10%), including difficulty in swallowing, limb weakness, seizure, facial palsy, and the necessity of receiving tracheostomy with or without mechanical ventilation. The mortality rate was 2.5% (10/401), and occurred primarily in patients with both encephalitis and cardiopulmonary failure.

**Table 1:** Demographic data and clinical outcome.

	Stage 1 Uncomplicated (N = 108)	Stage 2 Meningitis/ myoclonic jerk (N = 207)	Stage 3A Encephalitis (N = 45)	Stage 3B Encephalitis with cardiopulmonary failure (N = 41)	*P* value ^a^
**Age (yr)**	2.1 ± 1.0	1.9 ± 1.0	1.8 ± 1.1	1.4 ± 0.9	0.002
**Male/ Female**	69/39 (1.8)	120/87 (1.4)	25/20 (1.3)	30/11 (2.7)	0.233
**Weight (kg)**	12.8 ± 3.6	11.9 ± 3.2	11.3 ± 3.4	10.8 ± 3.5	0.004
**Hospitalization**	84 (78%)	201 (97%)	45 (100%)	41 (100%)	< 0.001
**ICU admission**	0 (0%)	21 (10%)	28 (62%)	41 (100%)	< 0.001
**Clinical Outcome**					
** Full recovery**	108 (100%)	207 (100%)	29 (64%)	6 (15%)	< 0.001
** Neurological Sequelae**	0 (0%)	0 (0%)	16 (36%)	25 (61%)	
** Death**	0 (0%)	0 (0%)	0 (0%)	10 (24%)	
**Galectin-3 SNP rs4644 genotype**					
** CC**	64 (59%)	140 (68%)	24 (53%)	29 (70%)	0.16^b^
** CA**	38 (35%)	59 (28%)	19 (42%)	11 (27%)	
** AA**	6 (6%)	8 (4%)	2 (4%)	1 (2%)	
**Allele frequency of rs4644**					
** C**	166 (77%)	339 (82%)	67 (74%)	69 (84%)	0.18
** A**	50 (23%)	75 (18%)	23 (26%)	13 (16%)	

^a^*P* value is determined by comparing the differences among 4 different severity groups.

^b^Logistic regression is used to analyze the association between clinical severity and rs4644 genotypes.

EV71 viremia predominantly occurs in children within the first three days of infection, and viral titers from throat swabs significantly declined subsequently [27]. Therefore, we analyzed throat EV71 viral titers during the first three days from the onset of clinical symptoms. Of 131 of 401 patients with viral culture obtained within 3 days of disease onset, the mean viral titers were as follows: AA genotype (2.7 ± 0.95 Log10 copies, N = 7), CC (3.0 ± 0.95 Log10 copies, N = 73), and CA genotypes (3.3 ± 1.00 Log10 copies, N = 51).

We further assessed whether rs4644 was associated with hyperglycemia in children with severe EV71 infection. At admission, 152 children had glucose examinations, and 39% (59/152) of them had hyperglycemia ≥ 150 mg/dL. We evaluated the mean serum glucose levels among children with different genotypes. Children carrying the AA genotype (2.2 ± 0.06 log_10_ mg/dL) had significantly lower blood glucose levels than those with the CC (2.4 ± 0.17 log_10_ mg/dL, *p* = 0.02) and CA (2.4 ± 0.15 log_10_ mg/dL, *p* = 0.03) genotypes, respectively. The leukocyte counts and CRP levels were similar among the three groups (data not shown). Among these hyperglycemic children, those with the AA genotype all fully recovered from the illness. However, the mortality rates were 15% (6/41) in children with the CC genotype and 7% (1/15) in those with the CA genotype. Neurology sequelae occurred in 43% (18/41) and 53% (8/15) of those with the CC and CA genotypes, respectively, and 0% (0/3) in those with AA genotype.

## Discussion

We have found that galectin-3 modulated EV71 replication via *in vitro* model. In addition, the A allele of rs4644, which mimics the findings in galectin-3 ablated cells, was associated with lower EV71 titers in our cellular model, and clinically the AA genotype was protective against higher levels of hyperglycemia, an indicator for severe EV71 infection. Both laboratory and clinical evidence suggest that the rs4644 A allele might exert a protective effect in EV71 infection.

Our study demonstrates that galectin-3 affects EV71 infection through three aspects. First, galectin-3 may modulate EV71 assembly, since the intracellular viral loads were not significantly different between RD and G3KO cells until 24 h of infection. Second, when apoptosis was inhibited, the total viral titers were decreased in RD cells, but the intracellular viral loads were not affected. In G3KO cells, both total and intracellular viral productions were not altered under the suppression of apoptosis (Fig 2B and S2C Fig). Our results suggest galectin-3 is required in EV71 release. Third, galectin-3 may modulate EV71 infection, partly through a caspase-independent pathway. As caspases were suppressed, our data showed the total and intracellular viral titers were still significantly higher in RD cells than in G3KO clones. EV71 can induce apoptosis through increasing endoplasmic reticulum (ER) stress and modification of mitochondrial activities [28–30]. Galectin-3 is also associated with mitochondrial functions and the expressions of the regulatory genes responsible for ER stress [31–33]. Thus, the role of galectin-3 in caspase-independent cell death in EV71 infection warrants further evaluation.

Regardless that we observed lower viral loads in RD cells expressing rs4644 A allele, such a difference in viral loads in clinical subjects cannot be ascertained due to inadequate statistical power (n = 7 for AA genotype), when the A-allele frequency is lower in Asian population compared with the Western population [34]. Nevertheless, the A-allele was indeed inversely associated with hyperglycemia, a surrogate for severe EV71 infection. Since the frequencies of CC genotype are much higher in Asians (58–75%) than Europeans (29%) [34], larger populations of C allele carriers may, in part, be responsible for EV71 outbreaks and severe cases in Asian countries.

Hyperglycemia is an important indicator of clinical severity in EV71 infection [1,5], as well as in other virus infections, e.g., influenza virus and respiratory syncytial virus. This is probably due to dysregulated systemic inflammatory responses, which lead to poor clinical outcomes [35–37]. In our cohort, we observed that the rs4644 AA genotype was associated with the lower serum glucose levels, but no differences in serum leukocyte counts and CRP levels, both indicators for inflammation severity. Dysregulated inflammatory responses cannot be excluded, since these data were obtained at single time point. Multiple sampling and concomitant examinations of cytokine changes are warranted to clarify the underlying mechanism. Moreover, EV71 replication affects glucose metabolism in the infected cells [38]. The interplay between EV71 and galectin-3 on the modulation of these glucose-regulatory proteins still requires further examination.

This study has several strengths. First, it provides a translational approach to examine the phenomenon obtained from functional assays in cell culture and the severity of diseases in clinical settings. Second, it supports the clinical association between galectin-3 SNP rs4644 and EV71-induced hyperglycemia, suggesting altered glucose metabolism might underlie clinical severity via mechanisms that involve galectin-3. Third, this is a large pediatric cohort of severe EV71 infection, in which more than 70% of the 401 subjects had stage 2-3B of EV71 infection. Nevertheless, the study has several limitations. First, the frequency of minor (A) allele of rs4644 was low in our cohort, as in other previously report in Asian cohorts [34], leading to a small number of subjects with the AA genotype. Certain differences between different genotypes were difficult to be compared due to inadequate statistical power. Second, most of our patients were recruited in tertiary hospitals. Clinical samples, e.g., throat viral titers, were obtained at a later time due to the lag during referral. Early biochemical specimens were harder to obtain, i.e. throat swab was obtained within 3 days only in 131 of the 401 patients. Third, most EV71 infection were self-limited and severe cases are relatively rare. Our findings might not apply to more general community-based EV71 infections. Nevertheless, the genotype distribution in our sample resembles the general population [34] and Hardy-Weinberg equilibrium is assumed; thus, our sample might still mimic the general population.

In conclusion, our study provides evidence that galectin-3 is important for EV71 infection and has an intricate role in glucose metabolism and disease severity in clinical settings. Whether the rs4644 variation effectively predicts disease severity in EV71 infection needs to be evaluated in larger studies in the future. Nevertheless, the role of galectin-3 in viral infection deserves more emphasis and warrants further investigation.

## Supporting Information

S1 Fig Galectin-3 ablation affects EV71 infection by suppressing viral release. (DOCX)

S2 Fig Galectin-3-ablated cells had lower activities of apoptosis. (DOCX)

S1 Method Caspase-3 ELISA. (DOCX)
