# Supplementary material for: Galectin-3 and Its Genetic Variation rs4644 Modulate Enterovirus 71 Infection
Source: PLoS One. 2016 Dec 21;11(12):e0168627. doi: 10.1371/journal.pone.0168627 (PMC5176291; doi:10.1371/journal.pone.0168627)

**Galectin-3 and its genetic variation rs4644 modulate enterovirus 71 infection**

Wen-Chan Huang, Hung-Lin Chen, Huan-Yuan Chen, Kuan-Po Peng, Yungling Lee, Li-Min Huang, Luan-Yin Chang, Fu-Tong Liu

**S1 Fig. Galectin-3 ablation affects EV71 infection by suppressing viral release.** Equal amounts of RD and G3KO cells were seeded in a 6-well plate. Cell numbers were measured by direct counting after 24 h of cell culture, and the numbers between RD and G3KO clones were not significantly different (A). RD and G3KO cells were seeded in 6-well plates. EV71 infection was performed (MOI 1) and viral titers were examined by plaque assays. Released EV71 viral loads at 8 and 24 h p.i. were evaluated by examining the viral titers in supernatant (B). The intracellular viral loads did not significantly differ between RD and G3KO clones at 3, 6, 9, 12 and 18 h p.i., which were determined by examining the viral titers of cell lysates (C). ***P < 0.001, n.s., not significant.


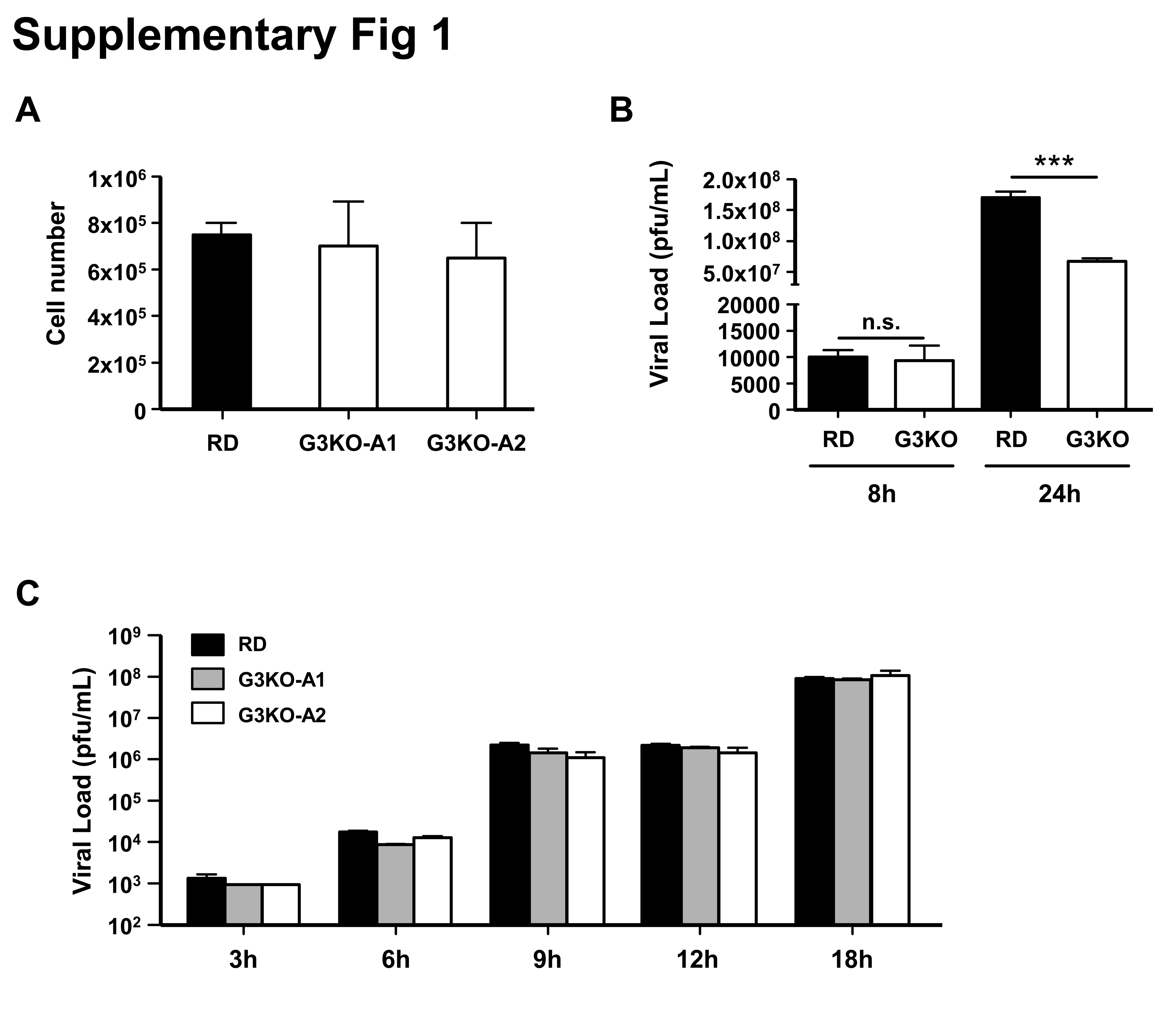

Supplement: S1 Fig — (DOCX) [file pone.0168627.s001.docx]
