# Supplementary material for: Galectin-3 and Its Genetic Variation rs4644 Modulate Enterovirus 71 Infection
Source: PLoS One. 2016 Dec 21;11(12):e0168627. doi: 10.1371/journal.pone.0168627 (PMC5176291; doi:10.1371/journal.pone.0168627)

**Galectin-3 and its genetic variation rs4644 modulate enterovirus 71 infection**

Wen-Chan Huang, Hung-Lin Chen, Huan-Yuan Chen, Kuan-Po Peng, Yungling Lee, Li-Min Huang, Luan-Yin Chang, Fu-Tong Liu

**S2 Fig. Galectin-3-ablated cells had lower activities of apoptosis.** RD and G3KO cells were infected with EV71 (MOI 0.1) and the cells were treated with or without a pan-caspase inhibitor, z-vad-fmk (50uM). Cell lysates were collected after 24 h of infection. The activities of apoptosis-related proteins, including PARP and caspase-3, were examined by immunoblots (A). Caspase-3 activities were examined by ELISA (B). The intracellular viral loads of RD and G3KO cells were measured by plaque assays (C). Casp3, caspase-3; LGALS3, galectin-3; ACTB, beta-actin; *P < 0.05; **P < 0.01.


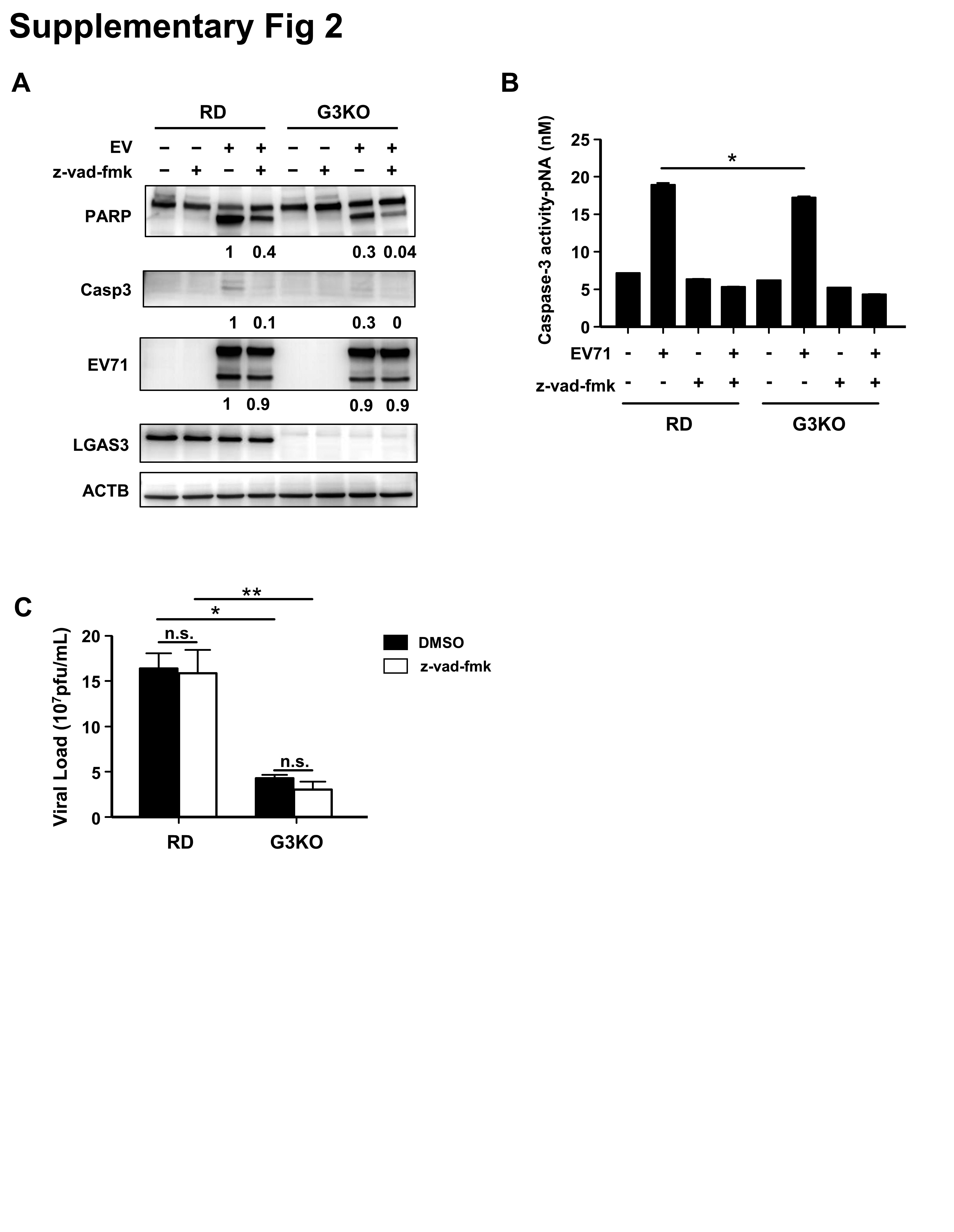

Supplement: S2 Fig — (DOCX) [file pone.0168627.s002.docx]
