# Supplementary material for: Galectin-3 and Its Genetic Variation rs4644 Modulate Enterovirus 71 Infection
Source: PLoS One. 2016 Dec 21;11(12):e0168627. doi: 10.1371/journal.pone.0168627 (PMC5176291; doi:10.1371/journal.pone.0168627)
Supplement: S1 Method — (DOCX) [file pone.0168627.s003.docx]

**Galectin-3 and its genetic variation rs4644 modulate enterovirus 71 infection**

Wen-Chan Huang, Hung-Lin Chen, Huan-Yuan Chen, Kuan-Po Peng, Yungling Lee, Li-Min Huang, Luan-Yin Chang, Fu-Tong Liu

**S1 Method. Caspase-3 ELISA.**

Cells were seeded in 96-well plates (4*10^4^ cell/well) and infected with EV71 (MOI 0.1). Cells were treated with or without a pan-casepse inhibitor, z-vad-fmk (50uM), during the entire course of infection. After 24 h of infection, viral supernatant was discarded and cells were washed with PBS for 2 times. Intracellular caspase-3 activities were determined by ApoAlert caspase-3 colorimetric assay kit (Clontech, Mountain View, CA, USA) according to the manufacturer’s protocol, with 4 replicates per cell types.
